# Perceptions of primary health care workers regarding violence against women

**DOI:** 10.1590/1980-220X-REEUSP-2021-0097

**Published:** 2022-01-24

**Authors:** Ariana Sofia Barradas da Silva, Mara Regina Santos da Silva, Deisa Salyse dos Reis Cabral Semedo, Daniela Claudia Silva Fortes, Alessandro Marques dos Santos, Kateline Simone Gomes Fonseca

**Affiliations:** 1 Universidade Federal do Rio Grande, Rio Grande, RS, Brazil.; 2 Universidade de Cabo Verde, Faculdade de Ciências e Tecnologia, Praia, Cabo Verde.

**Keywords:** Violence, Violence against women, Health Personnel, Women’s health, Forensic Nursing, Violencia, Violencia contra la mujer, Personal de Salud, Salud de la mujer, Enfermería Forense, Violência, Violência contra mulher, Profissionais de saúde, Saúde da mulher, Enfermagem Forense

## Abstract

**Objective:**

To identify the perceptions of Primary Health Care workers regarding Violence Against Women.

**Method:**

Qualitative, exploratory, descriptive study addressing 23 health professionals working in three Health Centers in Praia, Cape Verde, Africa. Semi-structured interviews were held via videoconference in November and December 2020. Data were treated according to thematic analysis.

**Results:**

Three categories emerged: violence against women restricted to physical aggression; violence as a phenomenon resulting from financial dependency; and victim blaming.

**Conclusion:**

The reductionist view of violence, as limited to physical harm, associated with financial dependency and victim blaming helps to unveil perceptions that ground the practice of health workers with women victims of violence and can support the planning of continuous education provided in Primary Health Care services.

## RESUMO

**Objetivo:**

Identificar a percepção dos profissionais que trabalham na Atenção Primária à Saúde acerca da Violência Contra Mulher.

**Método:**

Pesquisa qualitativa, exploratória, descritiva, desenvolvida com 23 profissionais de saúde que atuam em três Centros de Saúde da Cidade da Praia, Cabo Verde, África. Foram realizadas entrevistas semiestruturadas, por videoconferência, no período de novembro a dezembro de 2020. Os dados foram submetidos à análise de conteúdo na modalidade temática.

**Resultados:**

Foram identificadas três categorias: a violência contra a mulher circunscrita a agressão física; a violência como um fenômeno decorrente da dependência econômica; e a culpabilização da vítima pela situação de violência.

**Conclusão:**

A visão reducionista da violência delimitada ao dano físico, associada a fatores econômicos e culpabilização da vítima, ajuda a desvendar as percepções que embasam as práticas dos profissionais da saúde com mulheres vítimas de violência e serve de subsídios para o planejamento da necessária educação continuada nos serviços de Atenção Primária à Saúde.

## RESUMEN

**Objetivo:**

identificar la percepción de los profesionales, que trabajan en la Atención Primaria de la Salud, acerca de la Violencia Contra la Mujer.

**Método:**

investigación cualitativa, exploratoria y descriptiva, desarrollada en 23 profesionales de la salud, que actuaban en tres Centros de Salud en la Ciudad de Praia, Cabo Verde, África. Fueron realizadas entrevistas semiestructuradas, por video conferencia, en el período de noviembre a diciembre de 2020. Los datos fueron sometidos al análisis de contenido en la modalidad de la temática.

**Resultados:**

fueron identificadas tres categorías: la violencia contra la mujer circunscrita a la agresión física; la violencia como un fenómeno proveniente de la dependencia económica; y el sentimiento de culpa de la víctima por la situación de violencia.

**Conclusión:**

la visión reduccionista de la violencia limitada al daño físico, asociada a factores económicos y al sentimiento de culpa de la víctima, ayuda a revelar las percepciones en que se basan las prácticas de los profesionales de la salud con mujeres víctimas de violencia y sirve para auxiliar la planificación de la educación necesaria y continuada, en los servicios de Atención Primaria a la Salud.

## INTRODUCTION

Violence against women (VAW) has been a phenomenon present in history since old times and is a global concern given its magnitude and consequences on the lives of individuals, families, and communities. Therefore, it is a human rights violation, classified as an important public health problem, considered a priority in many countries’ public policy, and is part of the Sustainable Development Goals^(1)^.

Epidemiological data show that 30% of women worldwide have already been victims of intimate partner violence^(1)^. The same document reports that the highest rates are found in Southeast Asia (37.7%), followed by Eastern Mediterranean, with 36.6%. The African continent ranks third (36.6%), showing that, despite international, regional, and local efforts, including many conventions, treatises, and other measures to fight violence against women, it remains a recurrent and persistent phenomenon^(1)^.

Data from 2005 concerning Cape Verde, Africa, where this study was developed, show that more than one, in every five Cape Verdean women aged 15+, was a victim of one or various forms of violence perpetrated by an intimate partner. In 2015, in Praia, the country’s capital, the rate was 42.5%, and from 2018 onwards, femicide became frequent news in the national media^(2)^.

Regardless of the context, evidence from international literature describes VAW as a social, historical, and multifactor phenomenon that negatively impacts the victims’ health and qualify of life, compromising their social relationships and affecting the health system. In addition to the economic costs associated with the need to provide treatment and hospitalizations in mental health and emergency services, these women require social and legal support because victims are more likely to face unemployment, absenteeism, and loss of productivity due to factors such as long-term disabilities^(1,3)^.

In this context, Primary Health Care – PHC is a strategic instance to prevent, identify, and report violence, assist the victims, and coordinate care delivery. Therefore, it favors the connection between the health sector, education, social services, and the legal system, gathering the conditions necessary to provide integral health care, considering socioeconomic, social, family, community, individual, and gender factors that structure society^(4–5)^.

The insertion of PHC in the territories favors establishing a horizontal and dynamic dialogue with health care users, promoting lasting relationships. In addition, being close to users and specific programs directed to women’s health provides opportunities for workers to identify and act in cases of violence. However, despite these characteristics and the high prevalence of women dealing with violence in these services, detection rates are low, and health workers have difficulty acting in these situations for varied reasons^(4,6)^.

The reasons include the workers’ conceptions, principles, and values, which in many cases are developed in a culture impregnated by social and institutional standards that discriminate against women and consider violence to be a family problem. Additionally, out of fear from aggressors’ reprisals, workers may omit assistance, remain silent and not take responsibility for reporting violence, even though it is mandatory, while there is a lack of regular programs providing specific training in the services. As a result, professional practice is predominantly discriminatory and fragmented in this context, with interventions based on the clinical aspect, without fully considering the situation’s complexity^(7)^.

Considering that the workers’ perceptions shape their practice with women victims of violence and that such perceptions result from the interaction of many factors, this study was based on the Bioecological Theory of Human Development, specifically on the notion of context it presents. This notion refers to the interactions experienced by people in different environments, from the micro family context up to the macro context, in which the norms, cultural values, and gender roles are learned in the socialization process^(8)^. Additionally, this theoretical framework enables more deeply examining multi-dimensional phenomena such as VAW.

Therefore, this framework allows a better understanding of how workers perceive women victims of violence and their stand toward these situations. Therefore, this study’s objective is to identify the perceptions of PHC workers regarding violence against women and contribute to the qualification of the care provided in these services to victims.

## METHOD

### Design of Study

This is a exploratory, descriptive study with a qualitative approach.

### Population

Twenty-three female health workers aged between 24 and 55 participated in this study: 12 nurses, four (4) physicians, four (4) social workers, two (2) clinical psychologists, and one (1) nurse who is also a neuropsychologist. Note that the teams of the Health Centers hosting data collection are predominantly composed of women, which explains why only women composed the sample.

### Local

The study setting included three PHC Health Centers (HCs) in Praia, Ilha de Santiago – Cape Verde, Africa. The HCs are municipal facilities that provide primary health care to the population covered by their geographical area, ranging from 7,000 to 20,000 inhabitants^(9)^. Multidisciplinary teams coordinated by a physician ensure care is provided in the fields of prenatal care, childcare consultations, chronic ill patients, family planning, general practitioner consultations, nursing consultations, psychology, adolescent health, physical therapy, stomatology, and other specialties, such as dermatology, nutrition, and pediatrics^(9)^.

These services were chosen because they are located in areas presenting the highest rates of VAW complaints and concentrate most of the social structures involved in the fight against VAW, located near the PHC facilities.

### Selection Criteria

The professionals working in the services for at least six months were included in the study, except those on vacation, maternity leave, or away for other reasons.

### Data Collection

The primary author, a nurse, collected data during November and December 2020 through semi-structured interviews holding videoconferences between Brazil and Cape Verde, with the support of two research assistants in Cape Verde, where data were collected. All the authors integrate GEPEFES [Group for the Study and Research of Family Nursing and Health], one of its central thematic axes is domestic violence.

The script guiding the interviews initially addressed sociodemographic characteristics and then addressed the study objectives through the following guiding questions: “What is violence against women from your perspective?”, “What signs make you suspect that a woman is a victim of violence?”, “In your opinion, what are the reasons leading a woman to maintain a relationship with an aggressive partner?” and “In your opinion, what services or places are the most appropriate to provide care to women in situations of violence, and why?”

The first contact with the participants occurred in 2020 when the study’s objectives and the procedures used to collect data were explained. The interviews were scheduled in advance, and the participants were individually interviewed in a private room on the facility’s premises or at their homes. Interviews lasted 50 minutes on average and were recorded and transcribed verbatim.

### Data Analysis and Treatment

Data were organized and submitted to thematic content analysis according to the stages proposed by Minayo^(10)^: comprehensive and exhaustive reading, exploration of the material, treatment, and interpretation of data. First, each interview was read to identify the elements that indicated the workers’ perceptions of the phenomenon. Next, similarities and divergencies were identified in the content expressed in the participants’ reports, which were gathered into larger categories according to the study’s objectives. Finally, definitive categories were generated, and their defining elements were established. Next, we proceeded to interpret data based on the Bioecological Theory of Human Development^(8)^. This theoretical framework enabled the establishment of nexus between the way the workers perceive women victims of violence and contextual factors, including social, economic, and cultural factors.

### Ethical Aspects

This study was approved by the Institutional Review Board at the Federal University of Rio Grande, CAAE No. 37161420.2.0000.5324, and the National Research Ethics Commission, Ministry of Health and Social Security in Cape Verde, deliberation No. 59/2020. All ethical guidelines provided by Resolutions No. 466/2012 and No. 510/2016 regulating research involving human subjects were complied with^(11–12)^. A code was used to ensure the confidentiality of the participants’ reports. The letter “P” was followed by the number indicating the order in which each interview was held (P1, P2, P3…P23).

## RESULTS

### Violence Against Women Restricted to Physical Violence

Most of the participants revealed a perception that violence against women is mainly an act of physically abusing and controlling a woman, ignoring other manifestations of violence, such as psychological violence. In addition, physical violence was predominantly referred to as the easiest to recognize and solve, as the intervention is focused on treating body injuries. This is a restricted perception of violence against women that conditions its recognition and interventions to body injuries and somewhat exempts workers from investigating further other common complaints that emerge in PHC, masking other forms of violence.

*A woman comes in with a simple case of diarrhea. We carry out exams, and if we notice hematoma, we try to identify the cause. However, if she doesn’t present any signs, it may often go unnoticed* (P9).

*A young woman was seen at the service, and she had visible signs of aggression. So, they immediately called me, I welcomed her and offered support; this is generally our procedure* (P4).

Even though according to the reports, the interventions were limited, the participants were sensitive and indignant when they reported physical aggression against women, showing they were solidary to the victims. However, this engagement was not observed regarding other forms of violence against women. In these cases, they considered that the responsibility for actions lied with other services within the network.

*It is an unfortunate situation, I feel so sorry about these situations because I am a woman, and I appreciate my freedom and autonomy. It is difficult to accept that there are women who still suffer violence* (P8).

*It is shocking knowing that there are women deprived of their autonomy and are physically abused; there must be justice* (P17).

*I got so angry; she was all hurt, I applied the dressing and referred her* (P10).

Participants P5, P6, P7, and P12 refer that because the PHC service is located within the community, they often are acquaintances of the women abused by their partners. They also added that the community’s members often report these cases. However, the workers do not always take action in these situations for fear of being recognized or because women themselves are afraid of reporting.

*A woman came to the service and mentioned that her neighbor was abused by her partner; I made an active search but didn’t want to talk in detail at her home because her partner is very aggressive. So I asked her to seek me at the health service; she came but didn’t want to report…* (P5).

*I took care of a woman with a cut on her head. I already knew that she was abused; though, she told me it was an accident… A few days later, she came back and said her husband apologized and bought the groceries for the month. She asked me not to mention it to anyone* (P16).

The workers’ fear and perception that violence against women is restricted to physical violence may hinder preventive and protective actions among the women in the community, resulting in omitted care and miscommunication within the network.

### Violence as a Phenomenon Resulting from Financial Dependency

The health workers were unanimous in associating violence with financial dependency. This approach is one of the causes shaping interventions in health services, as they consider they will not be able to solve these women’s financial problems.

According to the participants’ reports, this phenomenon is evidenced in three situations. One is related to total financial dependency on the partner, who generally provides for the family. In this condition, women are afraid of not having the means of subsistence and letting their children unassisted; hence, they adapt to the situation. The second situation is associated with the woman having a precarious job and a low salary. Many women are not formally employed and depend on their partners to complement the family income and meet the family’s basic needs. The third situation is when the partner controls the woman’s money, and as a result, she ultimately becomes dependent on him.

*She doesn’t work and needs her husband’s money to support the children; hence, she stays in the relationship to prevent her children from going hungry* (P23).

*Even though women are currently more empowered, men are still the providers in many families, which enables them to have some power over them* (P9).

*There is a striking case of a 26-year-old lady whose husband doesn’t work. She is the one providing for her family; however, her husband spends part of her income in bars drinking with his friends, and when he gets back home, he beats his wife* (P20).

P8, P15, P17, and P19 note that financial dependency establishes a very complex connection. The participants reported that in an attempt to help these women, they refer them to social entities that can help them with basic supplies, food, hygiene products, and transportation. However, this process is often slow, and women opt for continuing with their partners as a way of subsistence for themselves and their children.

*Some of these women seek solutions, saying that financial support would help them get out of the situation. But, we do not offer this type of support here; we have a social worker, but this doesn’t work, it’s one-time support (…), so they give up* (P15).

In this context, health workers believe that the financial problem is not only a determinant for these women to remain in a situation of violence when the services do not readily meet their expectations, but it also contributes to them remaining longer in an abusive relationship.

### Victim Blaming

The reports of P1, P2, P3, P4, P5, P7, P8, P14, P18, and P21 reveal that they perceive women to be responsible for the situation of violence because they have a passive attitude toward it or consume alcohol. Therefore, it is up to these women to take the initiative and change their conditions. It is, therefore, a discourse that transfers responsibility to the women, and at the same time, exempts professionals from engaging in protective and preventive actions, which are their responsibility.

*In addition to being abused by her partner, she’s an alcoholic. After the report, we worked on a plan to help her, organized a structure, but she never came back; she just gave up. It’s challenging to work with an alcoholic* (P4).

*The woman won’t help; I referred her and everything; a few days later, I went on a home visit, went to the community, and she was with her husband; it’s complicated* (P18).

*Sometimes, the woman takes too long to talk about. Her partner mistreated her, but then, over time, she became confident and talked. We referred her, but then she returned and said she had sorted things out with her husband. I could see in her speech that it would happen again, it was a matter of time, but still, she went back* (P19).

When asked about the interventions implemented in other types of violence, besides physical violence, like psychological and patrimonial violence, the participants reported that it depends on the woman to recognize herself as a victim and report the fact so they can plan accordingly. Additionally, the participants report they are not prepared to provide other services due to their limitations or because these services are not under their competence.

*First, the woman needs to realize that she is suffering psychological violence; when reported, we have to refer her to psychological support or seek legal support* (P2).

*We tried to work with a psychologist, we tried everything, but it was extremely difficult to change her mindset because she thought it was a normal thing to happen* (P14).

*The woman has to accept care; even if I intervene, she has to allow it, let it flow within, and manage to get out of this cycle of violence* (P1).

When asked how women access PHC services, the participants say that when it comes to physical violence, women often seek the service or are referred by the police to treat their injuries. They also add that when women go to the policy department, they may occasionally be assisted by agents who hold a culturally machista view and naturalizes VAW, even asking them to reconcile with their partners. They also note that these women are generally referred to the PHC whenever they have a physical injury, making it difficult to provide proper care.

*The police officer assisting a woman can downplay the seriousness of the situation if he finds it to be something normal and expected (…) many times women verbalize that they are advised by these services to go back home and reconcile with their husbands (…) It may lead them to give up because they don’t feel these agents will provide the protection they need* (P8).

The participants’ reports reveal the influence of rooted cultural and social factors that challenge their competencies and resources to manage the situation appropriately.

## DISCUSSION

The reports addressed here portray the way these workers perceive the phenomenon. Even though the participants are from different fields and levels of work and years of experience vary, their conceptions regarding VAW are similar, in which physical violence predominates among the different forms of violence. Knowledge regarding other forms of VAW was superficial, and responses were limited. Similar results were found in a study conducted in Brazil, indicating a need to train workers because this superficial knowledge may compromise their assistance and the way they lead the process^(13)^.

This study’s findings show that the professionals consider violence an unacceptable situation that harms society. This perception may awaken workers’ motivation to act whenever they encounter a woman victim of violence. However, the results show an urgent need to train and prepare workers to deal with any form of VAW.

Likewise, the result of a review addressing qualitative studies highlights that the individuals’ belief systems can be shaped by personal experience or feminist ideological structures and human rights and define the intention of professionals to intervene whenever they face this situation, indicating that training by itself does not ensure effective care. Therefore, it is also necessary to focus on raising the awareness of these workers toward their conceptions^(14)^.

The purpose is not to change the workers’ perceptions because these are rooted in cultural factors that certainly require time. The suggestion here is to provide permanent education, presenting workers with the notion that there are other possibilities to make a difference in the lives of these women victims of violence. These actions work on multiple factors that influence the workers’ perceptions, possibly adding and contributing to their worldview in the medium and long term.

This study’s results are similar to the results reported by a study conducted in Japan, according to which recognizing women as victims of violence is usually easier when it involves physical signs and interventions are essentially based on the curative model^(15)^. In addition, the authors note that for workers to deal with these situations, communication and continuity of care are essential so that these situations can be identified and prevented at the beginning, avoiding it from getting worse.

As a result of care actions that are mainly focused on the curative model, studies show that women in situations of violence are at risk of experiencing worse mental health because violence changes their structural functioning and cause psychological conflicts; so that women found it challenging to make significant changes in their routines and break the cycle of violence^(16,17,18)^. In addition, psychological symptoms are associated with the violence itself and are a response to having their victimization disclosed with no effective solutions^(19)^.

In this context, this study highlights an urgent need for workers providing care to heed the psychological symptoms associated with violence. At the same time, there is a concern with the care provided to the victims because even though there are professionals available to meet the victims’ needs, care delivery may be compromised.

In the Cape Verdean context, this situation may be even more challenging because mental care has not been effectively integrated into care practice, and it may be difficult to diagnose mental disorders resulting from violence against women.

This study’s results show that financial dependency is a predictor of women remaining in a context of violence, which is consistent with studies that compare the perception of workers from different social contexts. For instance, Brazilian health and legal workers consider that financial dependency is the primary factor, while Norwegian workers report emotional dependency and social standards determining that families must remain united and children should not be exposed to divorce^(20)^. Another study shows that being financially dependent on the partner is the justification explaining why women are advised to reconcile with their partners^(21)^.

Hence, financial empowerment has been considered a protective factor against violence. Financial opportunities arising from planned interventions – such as microcredit and jobs created by non-governmental organizations, and education that enable women to assume new job positions or generate informal income, and family planning to free these women to generate income – are some of the mechanisms associated with decreased violence^(22)^.

These interventions have been implemented in Cape Verde. In this context, women are among the beneficiaries of financial empowerment social projects. There has also been an increased offer of mental health and reproductive services. However, these do not seem sufficient to avoid violence. These situations make us think that the financial issue *per se* is not a protective factor or what triggers violence. Instead, many interconnected factors may result in this outcome, arising from the various development contexts in which the individuals’ biopsychological characteristics interact with historical time.

The Cape Verdean context presents economic specificities. Even though the participants justified that women remained in violent relationships due to financial dependency, this situation requires careful analysis because official sources report that women are the primary providers responsible for supporting their homes. Women are the head and providers of their families and have the power over their households; however, they renounce this role and maintain marital relationships not because their partners support their families but because the presence of a man renders respect and social status^(23)^.

This study’s results suggest that women’s role usually results from social and historical constructs that mark their lives. Even if they have the resources to support themselves and manage their lives, the male figure determines how they behave, including what type of service they choose to care for their health. It is a matter to reflect upon if we consider that financially independent women allow their partners to control their lives; it must be even more challenging for women who are financially dependent on their partners. Hence, it leads us to infer that the determinant factor might not be financial dependency but gender issues, in which financial control is merely a manifestation of the latter.

As the participants reported, leaving an abusive relationship is particularly difficult because the cyclical nature of violence impacts the victims’ ability to perceive their actions as successful, decreasing their motivation to act. Hence, to cope with their relationships, these women tend to overestimate the positive aspects and hope for a change, which is one factor that determines how long women remain in these relationships. Therefore, breaking from an abusive relationship requires an individual to change her perceptions toward herself, her partner, and the relationship. Thus, the process of leaving a relationship is only possible by combining cognitive, interactional, financial, contextual, and social factors^(24)^, a view that holds women victims of violence responsible for protecting themselves against it.

The professionals’ interventions were justified by the involvement, availability, and disposition of women victims of violence to engage in the process. Additionally, the participants recognize that it is not an easy task, especially in highly sectoral and fragmented services, which may restrict their actions^(25)^.

Even though the respondents recognized men as the perpetrators of violence, they hinted that women are conniving and accept aggressive men as partners. The participants consider this to be an obstacle that hinders their interventions. Studies corroborate these findings, reporting attitudes and feelings of hopelessness toward male aggressors’ actions^(26)^.

Discourses supporting that women victims of violence are conniving with the situation make us wonder whether these health workers also share social and cultural standards that are tolerant of violence; it would be a situation that puts women in an even greater vulnerability.

This study’s findings highlight that the care provided to women victims of violence is based on curative care and referral to other services. These results are consistent with some studies reporting that the professionals tend to treat the symptoms without addressing the cause because they often fail to recognize other factors associated with violence, providing fragmented care. At the same time, little or no collaboration is established with other sectors^(27–28)^.

This study addressed professionals working in a specific and restricted context, which prevents data generalization. Additionally, only female workers participated in this study, so it was impossible to capture the perceptions of male workers.

It would be interesting to address the perceptions of the women victims of violence and those of the community where the service is located. Integrating these perceptions would enable understanding the phenomenon’s complexity; hence, we suggest studies are conducted with the women victims of violence using these services.

## CONCLUSION

This study reveals a reductionist view of violence restricted to physical harm, associated with financial dependency and victim blaming, which ground the practices of workers providing care to women victims of violence. Note that there is little collaboration between the PHC services and among the workers integrating the Health Centers teams, compromising the protection of these women and even exposing them further, by letting them deal with often mutilating situations by themselves, even though they are the users of services supposed to protect them.

This study’s results are expected to support continuing education actions in Primary Health Care and encourage reflections among the professionals in these services, contributing to qualifying the care provided to women victims of violence.

## Financial support

This work was carried out with the support of the Coordenação de Aperfeiçoamento de Pessoal de Nível Superior – Brazil (CAPES) – Financing Code 001 – DS Masters Scholarship granted to Ariana Sofia Barradas da Silva.
